# Emergence of a Novel Pathogenic Poxvirus Infection in the Endangered Green Sea Turtle (*Chelonia mydas*) Highlights a Key Threatening Process

**DOI:** 10.3390/v13020219

**Published:** 2021-01-31

**Authors:** Subir Sarker, Christabel Hannon, Ajani Athukorala, Helle Bielefeldt-Ohmann

**Affiliations:** 1 Department of Physiology, Anatomy and Microbiology, School of Life Sciences, La Trobe University, Melbourne, VIC 3086, Australia; a.athukorala@latrobe.edu.au; 2 School of Veterinary Science, University of Queensland, UQ Gatton Campus, Gatton, QLD 4343, Australia; c.hannon1@uq.edu.au (C.H.); h.bielefeldtohmann1@uq.edu.au (H.B.-O.); 3 Australian Infectious Diseases Research Centre, The University of Queensland, St. Lucia, QLD 4072, Australia

**Keywords:** green sea turtle, poxvirus, skin lesions, species conservation

## Abstract

Emerging viral disease is a significant concern, with potential consequences for human, animal and environmental health. Over the past several decades, multiple novel viruses have been found in wildlife species, including reptiles, and often pose a major threat to vulnerable species. However, whilst a large number of viruses have been described in turtles, information on poxvirus in cheloniids remains scarce, with no molecular sequence data available to date. This study characterizes, for the first time, a novel poxvirus, here tentatively designated cheloniid poxvirus 1 (ChePV-1). The affected cutaneous tissue, recovered from a green sea turtle (*Chelonia mydas*) captured off the Central Queensland coast of Australia, underwent histological examination, transmission electron microscopy (TEM), DNA extraction and genomic sequencing. The novel ChePV-1 was shown to be significantly divergent from other known poxviruses and showed the highest sequence similarity (89.3%) to avipoxviruses (shearwater poxvirus 2 (SWPV2)). This suggests the novel ChePV-1 may have originated from a common ancestor that diverged from an avipoxvirus-like progenitor. The genome contained three predicted unique genes and a further 15 genes being truncated/fragmented compared to SWPV2. This is the first comprehensive study that demonstrates evidence of poxvirus infection in a marine turtle species, as well as a rare example of an avipoxvirus crossing the avian-host barrier. This finding warrants further investigations into poxvirus infections between species in close physical proximity, as well as in vitro and in vivo studies of pathogenesis and disease.

## 1. Introduction

The green sea turtle (*Chelonia mydas*) is categorized as a “vulnerable” species in Australian waters [1], and “endangered” globally [2]. It plays an integral role ecologically, culturally and economically and, in recent decades, the species has been the focus of intense population monitoring and conservation efforts. The green sea turtle may also be considered an excellent sentinel species, with their health indicative of the health of their local ecosystems, due to their high site fidelity in their post-pelagic neritic feeding grounds [3,4,5]. At 5–10 years of age, pelagic juvenile green sea turtles return to coastal environments and establish a feeding site, where their diet consists predominantly of macroalgae, seagrass and mangrove material [6,7,8]. Their value as a sentinel species is particularly relevant in Gladstone Harbor, Australia, where feeding green sea turtles display an average cumulative home range of only 6.7 ± 0.8 km^2^ 95% kernel utilization distribution [9]. The Gladstone Harbor estuary, located at the southern end of the Great Barrier Reef Marine Park, supports critical marine animal and migratory bird habitat, with expansive seagrass meadows and intertidal wetlands [10]. However, the harbor is also home to an urban center, recreational and commercial fishing, and major industrial operations. In fact, it is the fourth largest multicommodity port in Australia, with rapid expansion in the past five decades alongside the coal, alumina and liquefied natural gas industries (LNG) in Central Queensland. In order to monitor and assess the potential anthropogenic pressures this industry may have on the local ecosystem, and furthermore the Great Barrier Reef, investigations into the causes of morbidity and mortality in the large feeding population of green sea turtles within the port have been conducted. Considered causes of disease should include infectious pathogens, including viruses.

The role of viral infections in marine turtle pathology is currently poorly understood. It is, however, a burgeoning field of investigation. There have been significant advancements in the study of cheloniid alphaherpesvirus 5 (ChHV5) and associated host–viral dynamics [11,12,13], along with the discoveries of *Chelonia mydas papillomavirus* (CmPV) [14,15] and sea turtle tornovirus 1 (STTV1) [16], and their potential roles in systemic disease and fibropapillomatosis. However, the green sea turtle virome is likely far more diverse and complex than currently accounted for, with potentially significant implications for host immune status and disease. This report characterizes a novel poxvirus infection in a green sea turtle and, in the context of conservation and disease monitoring, represents a significant contribution to the scientific community’s knowledge of sea turtle virology.

Poxviruses are large, enveloped, double-stranded DNA (dsDNA) viruses, typically affecting the integumentary system with eosinophilic intracytoplasmic inclusion bodies often seen in infected cells. Based on their host range, the family *Poxviridae* is currently divided into two subfamilies, namely *Entomopoxvirinae* and *Chordopoxvirinae*, which infect insects and vertebrates, respectively [17]. The *Chordopoxvirinae* subfamily contains 18 genera, many of which have been studied in-depth due to their medical and veterinary relevance [18]. However, relatively little is known about the origins, worldwide host distribution and genetic diversity of the poxviruses that infect reptilian species. Currently, the only accepted genus known to infect reptiles is the *Crocodylidpoxvirus* genus, known to infect many crocodilian reptile species. It is represented by the Nile crocodile poxvirus (CRV) first isolated from Nile crocodiles (*Crocodylus niloticus*) [19], and the still-unassigned saltwater crocodilepox viruses, first isolated from Australian saltwater crocodiles (*Crocodylus porosus*) [20,21]. Poxviruses have also been suspected of infecting other reptile species, in most cases based on gross lesions or intracytoplasmic inclusions seen on electron microscopy; these include Hermann’s tortoise (*Testudo hermanni*) [22], the flap-necked chameleon [23], the tegu lizard (*Slavator merianae*) and the desert tortoise (*Gopherus agassizi*) [24]. To this date, there has been only one report of a poxvirus-like infection in a cheloniid, the softshell turtle (*Pelodiscus sinensis*), which was again suspected based on gross lesions, histology and transmission electron microscopy (TEM). This report represents the first green sea turtle poxvirus infection described in scientific literature, as well as the first example of a non-*Crocodilia* reptile poxvirus infection confirmed by whole-genome sequencing.

## 2. Materials and Methods

### 2.1. Animal Capture and Tissue Sampling

The affected wild green sea turtle was captured on the 3 November 2017 in a manned, 300 m long blocking net in Gladstone Harbor, QLD (GPS −23.76624, 151.30533) by the Queensland Government Department of Environment and Science (DES). The turtle was included in a green sea turtle survey as part of the Gladstone Ports Corporation Ecosystem Research and Monitoring Program (ERMP) and the Long-Term Turtle Monitoring Project (LTTMP). It was transported 11 km by boat to a processing facility on land, however, on arrival it was found to be moribund, and although it was initially successfully resuscitated, died approximately 12 h later. Morphometric data collected included curved carapace length (CCL) of 93.0 cm and body weight of 104.5 kg and confirmed the turtle to be an adult female in excellent body condition. During post-mortem examination, multiple samples of cutaneous tissue were taken from the posterior fore flippers with a sterile scalpel and placed immediately in 10% neutral-buffered formaldehyde for further analysis.

Turtle handling, sampling and research activities were undertaken in accordance with the standard practices approved under the DAFF Animal Experimentation Ethics Committee: Queensland Turtle Conservation Project SA 2018-11-660, 661, 662, 663, 664. The use of nets for the capture of turtles was in accordance with DAF General Fisheries Permit 191182, issued to EHP/DES. The studies also received approval from the University of Queensland Animal Ethics Committee (permit no. AE43201).

### 2.2. Histology

The tissue samples were routinely processed for paraffin embedding and 5 µm sections, stained with eosin and hematoxylin (H&E) and examined on an Olympus microscope.

### 2.3. Transmission Electron Microscopy (TEM)

A small piece of paraffin-embedded sample was removed from the relevant region and placed in xylene overnight. It was then rehydrated through a graded ethanol series and placed into 1% osmium tetroxide as a post fixative. After washing in water, it was rehydrated through ethanol and embedded in Epon resin. Ultrathin sections were cut on a Leica Ultracut UC6 ultramicrotome and picked up on copper grids. These were stained with uranyl acetate and lead citrate and observed in a Hitachi HT7700 transmission electron microscope.

Histologically suspected cutaneous tissue materials were suspended 1:10 in phosphate-buffered saline (PBS), homogenized, clarified and adsorbed onto 400-mesh copper EM grids, before staining and imaging on a JEOL JEM-2100 transmission electron microscope as previously described [21,25].

### 2.4. Extraction of DNA

A formaldehyde-fixed sample of cutaneous tissue was sent to La Trobe University for molecular investigation. There, it was aseptically dissected and mechanically homogenized in lysis buffer followed by total genomic DNA being isolated according to the published protocols [26,27] using a ReliaPrep gDNA Tissue Miniprep System (Promega Corporation, Madison, Wisconsin, USA).

### 2.5. Library Construction and Sequencing

A total of 10 ng of extracted genomic DNA was used to prepare the library of 150-bp paired-end using the protocol adapted previously using a QIAseq FX DNA Library Kit (Qiagen, Germantown, MD, USA) [28]. The quality and quantity of the prepared library was assessed using an Agilent Tape Station (Agilent Technologies, Mulgrave, VIC, Australia) by the Genomic Platform, La Trobe University. The prepared library was sequenced on Illumina^®^ NextSeq 500 (Illumina, San Diego, CA, USA) platform according to the manufacturer’s instructions through the Australian Genome Research Facility, Melbourne.

### 2.6. Genome Assembly

The resulting 19.7 million raw sequence reads from NextSeq 500 were used to obtain the complete genome of ChePV-1 as per protocol described previously [25,28,29] using CLC Genomics Workbench (version 9.5.4, CLC bio, a QIAGEN Company, Prismet, Aarhus C, Denmark) and Geneious (version 10.2.2, Biomatters Ltd., Auckland, New Zealand). All raw sequencing reads were evaluated for quality and preprocessed to remove ambiguous base calls and poor quality reads and trimmed to remove the Qiagen Universal adapter sequences. Potential host DNA contamination was removed by mapping of trimmed sequence reads against the Chinese softshell turtle (*Pelodiscus sinensis*, accession no. GCA_000230535). Moreover, reads were filtered against *Escherichia coli* bacterial genomic sequence (GenBank accession no. U00096) to remove possible bacterial contamination. Unmapped reads were used as input data for de novo assembly using SPAdes assembler (version 3.10.1) [30] under the “careful” parameter in LIMS-HPC cluster (La Trobe Institute for Molecular Science—High Performance Computing cluster, specialized for genomics research in La Trobe University). This resulted in the generation of a 343,132-bp ChePV-1 genome obtained from a green sea turtle. A total of 16.62 million clean raw reads were mapped back to the genome sequence using CLC Genomics Workbench (version 9.5.4) that resulted in average coverage of 2849.85 X (minimum coverage = 5, maximum coverage = 11,368, standard deviation = 928.18).

### 2.7. Genome Annotation and Bioinformatics

The assembled ChePV-1 genome was first annotated using the Genome Annotation Transfer Utility (GATU) [31], where shearwaterpox virus (SWPV2) genome (GenBank accession no. KX857215) was used as the reference genome and further verification of the predicted open reading frames (ORFs) were performed using Geneious (version 10.2.2). The following criteria were selected to annotate as potential open reading frames (ORFs): (i) ORF longer than 50 amino acids, (ii) overlaps cannot exceed 50% of one of the genes to other ORFs, (iii) with an exception, ORFs <50 amino acids that indicated potential poxviruses orthologs and previously annotated in other poxvirus genomes. These ORFs were subsequently extracted into a FASTA file, and similarity searches including nucleotide (BLASTN) and protein (BLASTX and BLASTP) were performed on annotated ORFs as potential genes if they shared significant sequence similarity to known viral or cellular genes (BLAST E value ≤ 1.0 × 10^−5^) or contained a putative conserved domain as predicted by BLASTX and BLASTP [32].

Unique ORFs predicted in this study were further analyzed using multiple applications to identify conserved domains or motifs. Transmembrane (TM) helices were searched using the TMHMM package v.2.0 (DTU Health Tech, Lyngby, Denmark) [33], HMMTOP [34], TMpred [35] and Geneious (version 10.2.2, Biomatters Ltd., Auckland, New Zealand). Additionally, searches for conserved secondary structure (HHpred) [36] and protein homologs using Phyre2 [37] and SWISS-MODEL [38] were used. We also used SignalP v.5.0 [39] to identify possible signal peptides.

### 2.8. Comparative Genomics

The genetic organization of the newly assembled ChePV-1 genome with SWPV2 was visualized and compared using Geneious (version 10.2.2). Pairwise identity of the representative ChPVs species against ChePV-1 on the basis of complete genome nucleotide sequences were calculated using Base-by-Base software [40]. Selected concatenated proteins sequences identity was calculated in Geneious (version 10.2.2). Dot plots were created based on the EMBOSS dottup program in Geneious software, with word size = 12 [41].

### 2.9. Phylogenetic Analyses

Phylogenetic analyses were performed using the novel ChePV-1 genome sequence determined in this study together with other selected ChPVs genome sequences available in the GenBank database. Based on ChePV-1 genome sequence similarity from an initial BLAST search, related genome sequences of each of the fully sequenced ChPVs (Table 1), were downloaded from GenBank and used in further analysis for cheloniid poxvirus 1 (ChePV-1; MT799800) sequenced in this study. Nucleotide sequences of the selected complete genome of ChPVs and concatenated amino acid sequences of the selected nine poxvirus core proteins were aligned as described previously [28] with MAFTT (version 7.450) using G-INS-i (gap open penalty 1.53; offset value 0.123) algorithm implemented in Geneious (version 10.2.2, Biomatters, Ltd., Auckland, New Zealand) [42]. For the maximum likelihood (ML) tree, the program jModelTest 2.1.3 favored a general-time-reversible model with gamma distribution rate variation (GTR + G4) [43]. Phylogenetic analysis for concatenated amino acids sequences was performed with 1000 bootstrap support in Geneious (version 10.2.2). Analyses of non-tree-like evolutionary relationships amongst selected ChPVs were performed using NeighbourNet Bootstrap (1000 replicates) under default parameters in SplitsTree4 [44].

## 3. Results

### 3.1. Gross Lesions

There was dark purple discoloration of the skin of the posterior margins of all four flippers, along with the ventral skull, neck and tail. The dorsal surfaces of the fore flippers were unusually smooth. Multiple other pathologies were found on necropsy and histology, including changes consistent with pancreatitis, cholangiohepatitis, vasculitis, endocarditis, myocarditis, renal calculi, nephritis, cystitis.

### 3.2. Histology and TEM

**Figure 1:**
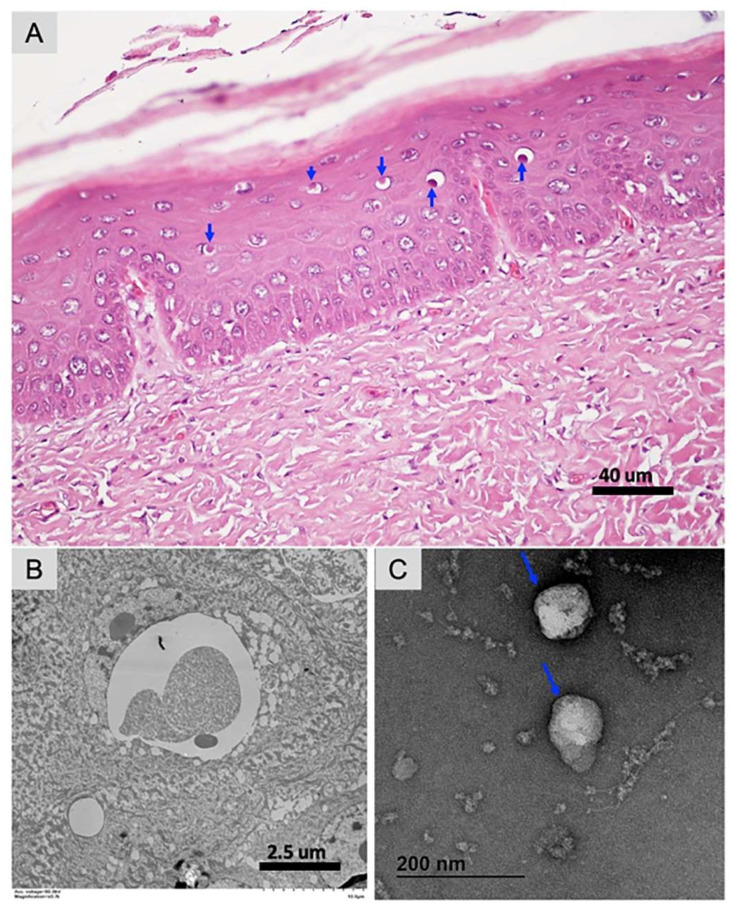
Pathological and transmission electron microscopic analysis of cutaneous tissue collected from a wild green sea turtle. (**A**) Histological changes are characterized by vacuolation and intracytoplasmic eosinophilic inclusions in cells in the stratum spinosum with frequent displacement of the nucleus to the periphery (blue arrows). H&E stain, scale bar = 40 µm. (**B**) Transmission electron microscopy (TEM) on tissue section, no viral particles were discerned in these inclusions, which appeared to be composed of proteinaceous material. Scale bar = 2.5 µm. (**C**) Cheloniid poxvirus particles showing brick-shaped virion with regularly spaced thread-like ridges comprising the exposed surface, measuring approximately 140 nm × 98 nm. Outer envelope of cheloniid poxvirus particles is highlighted with a blue arrow.

Histologically, the epidermis was characterized by focal occurrence of vacuolated cells in which eosinophilic inclusions were apparent (blue arrows in Figure 1A). By transmission electron microscopy (TEM) no viral particles were discerned in these inclusions, which appeared to be composed of proteinaceous material (Figure 1B). However, TEM of extracted material from the skin lesions revealed brick-shaped, poxvirus-like particles with regularly spaced thread-like ridges comprising the exposed surface and a visible outer envelope. The particles measured approximately 140 nm in length × 98 nm in width (Figure 1C), which was relatively small compared to poxviruses of other vertebrates and insects. The size and shape of the poxviruses detected in clinical samples can vary. For example, fowlpox virus propagated in baby hamster kidney can be large as 270 × 350 nm detected by negatively stained EM [52]. Recent studies reported that the size and shape of two avipoxviruses detected in clinical samples varies; a magpiepox virus particle was brick-shaped, measuring approximately 243 × 135 nm [28] and a mudlarkpox virus particle was brick-shaped to ovoid, measuring approximately 210 × 172 nm in diameter of larger virus particle [53].

### 3.3. Genome Structure and Analysis of ChePV-1

The assembled cheloniid poxvirus 1 (ChePV-1) complete genome was a linear double-stranded DNA molecule of 343,132 bp in length. Most chordopoxviruses (ChPVs) that infect vertebrates have genomes ranging from 133 to 360 kbp. ChePV-1 was more closely related to avipoxvirus than any other ChPVs, by virtue of genome length, genomic structure, complete genome sequence similarity and A + T content. For example, the ChePV-1 genome contained a large central coding region bound by two identical inverted terminal repeat (ITR) regions. The length of the ITR varies considerably in other poxviruses and ranges from <0.1 to 12.4 kb [54]. The ITR of ChePV-1 comprised 4118 bp each and coordinates 1–4118 sense orientation and 339,015–343,132 antisense orientation. Each of the inverted repeats constitutes arrays of direct repeats, and thirteen tandem repeats were detected within each inverted terminal repeat region, which consisted of a 155 bp, two 41 bp, a 24 bp, two 17 bp, two 16 bp, and five 9 bp repeat units and sharing approximately 71–100% nucleotide identity. Similar to most other poxvirus genome sequencing projects, hairpin loops were not detected, but it is believed that the ChePV-1 genome sequence included all coding regions because the final regions of the ITRs are a series of repeats. The A + T content of the complete genome sequence of ChePV-1 was found to be 71.6%, which is also comparable to other avipoxviruses [51]. The known poxvirus genomes that were most closely related to the ChePV-1 according to phylogenetic analysis were shearwaterpox virus-1 (SWPV1) (72.4% A + T), magpiepox virus (MPPV) (71.4% A + T), SWPV2 (69.8% A + T) and canarypox virus (CNPV) (69.6% A + T) [28,29,51].

**Figure 2:**
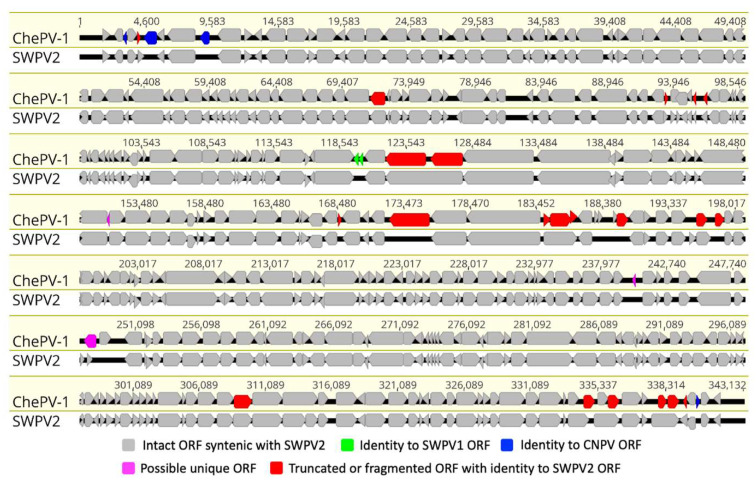
Comparative genomic illustration of the novel Cheloniid poxvirus 1 (ChePV-1) with shearwater poxvirus-2 (SWPV2). Sequence alignment using MAFFT in Geneious (version 10.2.2) was performed to compare open reading frames (ORFs) between cheloniid poxvirus (ChePV-1, GenBank accession no. MT799800) and shearwater poxvirus-2 (SWPV2, GenBank accession no. KX857215). The arrows symbolize genes and open reading frames (ORFs), indicating their direction of transcription. Each gene or ORF is color-coded, as indicated by the color key in the legend.

The ChePV-1 genome contained 329 predicted methionine-initiated ORFs encoding proteins which have been annotated as putative genes and were numbered from left to right (Figure 2 and Supplementary Table S1). Among them, four predicted ORFs were located within the inverted terminal repeats (ITRs), and accordingly were present as diploid copies. Comparative analyses of the predicted ORF sequences were performed and significant protein homologs for 326 ORFs of ChePV-1 were found (E value ≤ 1.0 × 10^−5^) (Supplementary Table S1). Moreover, ChePV-1 contained three predicted protein-coding genes that were not present in any other poxvirus, nor did they match any sequences in the NR protein database using BLAST search. These unique ORFs encoded proteins 53 to 65 aa in length (Supplementary Table S1). Interestingly, three novel ORFs (ChePV-1-134, −220 and −227) were predicted to contain transmembrane helices (TMHs) by several programs used in this study, but no classical signal peptide. ORF134 and −220 were predicted to contain one transmembrane helix in each, whereas ORF227 contained two transmembrane helices (Supplementary Table S1). Nonetheless, there was no evidence for conserved secondary structure, and no protein homologs were detected by various software, including HHpred [36], (Phyre2) [37] and SWISS-MODEL [38].

At the center of the ChePV-1 genome, all the ChPVs-specific conserved genes that were methionine-initiated, encoding ORFs were predicted (Figure 2 and Supplementary Table S1). The central region of the ChePV-1 genome appeared to be relatively highly conserved in gene content and synteny compared to the recently isolated shearwater poxvirus-2 (SWPV2) (Figure 2). Among these conserved chordopoxvirus gene products, the highest number of protein-coding genes (320) in ChePV-1 showed homology with SWPV2. The remaining four gene products (ORF03, −06, −09 and −327) were homologous with ORFs of CNPV, and a further two gene products were homologous with SWPV1 (ORF117 and −118) (Supplementary Table S1). Notably, in comparison to SWPV2, four gene products were missing in the ChePV-1 genome, and a further 15 genes were found to be truncated/fragmented (Figure 2 and Supplementary Table S1).

**Figure 3:**
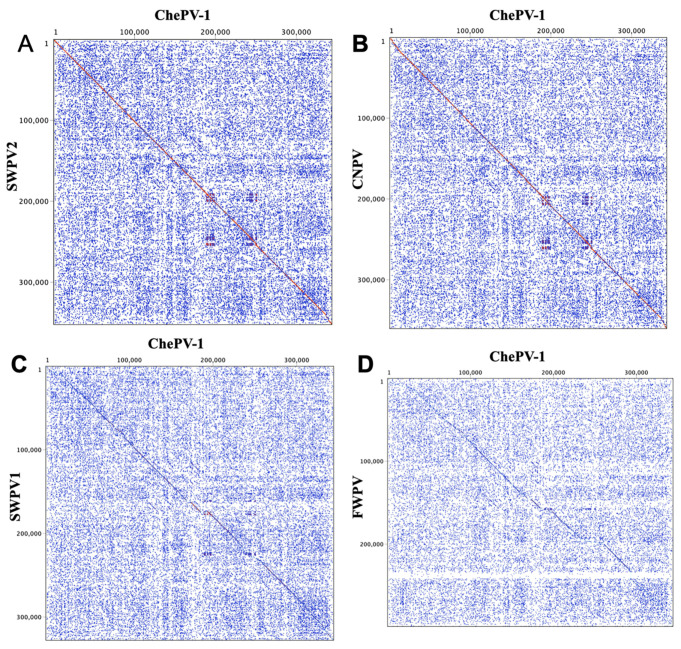
Dot plots of the ChePV-1 genome (x-axis) vs. other selected sequenced poxvirus genomes (y-axis). (**A**) ChePV-1 vs SWPV2, (**B**) ChePV-1 vs canarypox virus (CNPV) (**C**) ChePV-1 vs shearwaterpox virus-1 (SWPV1) and (**D**) ChePV-1 vs fowlpox virus (FWPV). The Classic color scheme was chosen in Geneious (version 10.2.2) for the dot plot lines according to the length of the match, from blue for short matches, to red for matches over 100 bp long. Window size = 12.

Based on multiple genome alignment using Base-by-Base, ChePV-1 was shown to be 89.30%, 88.86%, 86.83%, 75.56%, 66.16%, 64.70%, 64.41%, 64.35%, 63.18% and 39.02% identical (nt) to SWPV2, CNPV, MPPV, SWPV1, fowlpox virus (FWPV), penguinpox virus (PEPV), flamingopox virus (FGPV), pigeonpox virus (FeP2), turkeypox virus (TKPV) and crocodylidpoxviruses (e.g., CRV and saltwater crocodile poxvirus (SwCRV)), respectively. Similar to what we expected from percentage identity among selected chordopoxviruses genome, dot plots analysis showed the ChePV-1 genome to be highly syntenic with SWPV2 and CNPV (Figure 3A,B), and demonstrated major differences compared to SWPV1 and FWPV (Figure 3C,D).

### 3.4. Evolutionary Relationships of ChePV-1

**Figure 4:**
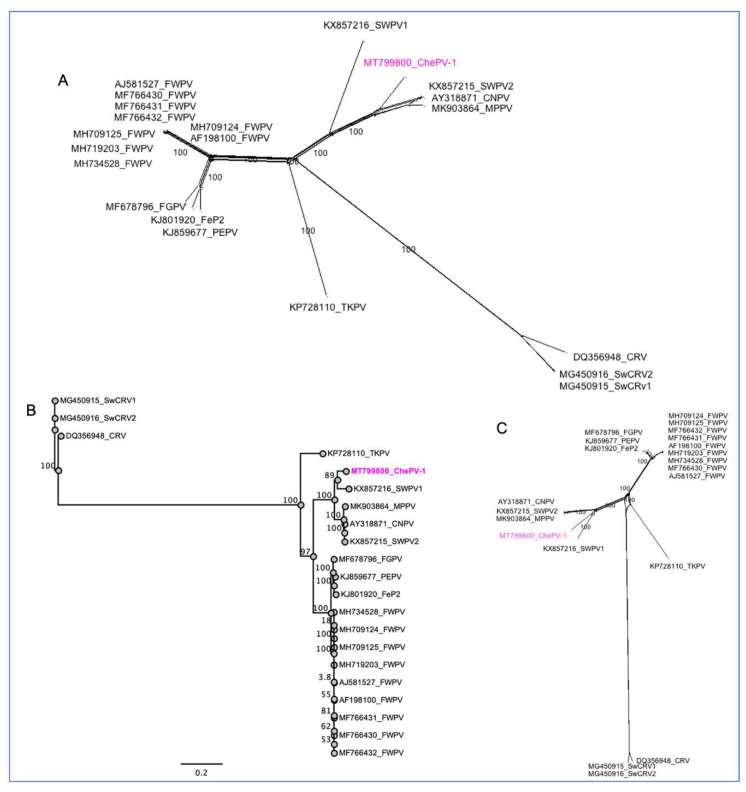
Phylogenetic relationship between novel cheloniid poxvirus 1 (ChePV-1) and other chordopoxviruses. (**A**) Bootstrap Network based on the complete genome sequences of the selected ChPVs. (**B**) Maximum likelihood (ML) tree and (**C**) Bootstrap Network based on the concatenated amino acid sequences. The numbers on the left show bootstrap values as percentages, and the ChePV-1 is highlighted with magenta color. The labels at branch tips refer to original ChPVs GenBank accession number followed by abbreviated species name.

Phylogenetic analyses using selected full-genome sequences of ChPVs and concatenated amino acid sequences of nine selected conserved ChPVs genes supported the inclusion of the newly assembled ChePV-1 as a likely avipoxvirus. In the resulting tree, based on complete genome sequences of ChPVs, the novel ChePV-1 was positioned in a distinct subclade (bootstrap support 100%) with CNPV with the recently isolated SWPV2 and MPPV, demonstrating that the ChePV-1 likely originated from a common ancestor of these viruses (Figure 4A). By also building phylogenetic trees using concatenated amino acid sequences of nine selected conserved ChPVs genes, we observed that the novel ChePV-1 placed with SWPV1 in a distinct subclade with strong bootstrap support (89%) (Figure 4B,C). Using the same set of concatenated protein sequences, the maximum inter-subclade sequence identity values between the novel ChePV-1 and other poxviruses were >91.70%, 91.29%, 91.26% and 91.21% (SWPV1, CNPV, SWPV2 and MPPV, respectively). There was no close relationship phylogenetically nor at sequence identity level with the sequenced poxviruses of reptilian origin (e.g., CRV and SwCRV).

## 4. Discussion

The findings of this report confirm this green sea turtle was infected with a poxvirus, ChePV-1. Applying various approaches in this study, the ChePV-1 genome was found to be genetically novel when compared to currently known poxviruses in the NCBI database. It showed highest sequence similarity with avipoxviruses, in particular SWPV2 (89.30%) and CNPV (88.86%); GC content of ChePV-1 (29.4%) was also comparable with that of avipoxviruses. Given this phylogenetic relationship, the authors postulate that this ChePV-1 originated from a common ancestor that diverged from an SWPV-like progenitor related to shearwaterpox and canarypox viruses.

Interestingly, ChePV-1 was shown to be highly genetically divergent from poxviruses that infect other reptilian species, such as CRV in Nile crocodiles and SwCRV-1 and −2 in saltwater crocodiles, with a GC content of 62% in all three reptilian viruses further supporting this divergence [19,20,21]. These phylogenetic relationships of ChePV-1 are unexpected findings considering that avipoxviruses tend to show a high degree of host–virus codivergence, typically with host specificity expressed at the level of phylogenetic order [55]. A search of the literature found only one similar example; that of an avipoxvirus isolated from a terminally ill rhinoceros in 1969 and characterized as an atypical fowlpox virus [56]. However, there are other genera of poxviruses that contain examples of species able to infect a broader range of hosts within a phylum; vaccinia virus and monkeypox virus, which have been associated with outbreaks involving humans and cattle [57,58], and cowpox virus which infects at least 27 species, including humans, cattle, equids and felines [18,59]. The findings in this paper indicate the avipoxviruses may have a broader host range and ability to cross host barriers than originally thought.

It is not possible to elucidate the host–pathogen dynamics of ChePV-1 from this case alone, but it is evident that marine turtles and avian species share an ecological niche to some extent. Port Curtis supports a large and diverse population of avian species [60]. The location where this turtle was captured, Pelican Banks at the South-East end of Curtis Island QLD, contains extensive *Zostera muelleri* seagrass meadows, large intertidal flats, mangrove networks and rocky shores [10,61] that are utilized as the feeding grounds for a large population of green sea turtles. The same habitats at Pelican Banks are host to internationally important numbers of roosting and foraging migratory shorebirds [62]. Both groups display a high level of foraging site fidelity [9], with green sea turtles in most cases only leaving the area for reproductive activities; examination of the ovaries in this individual indicated it had not bred for at least three years, indicating that avipoxvirus transmission likely occurred within the vicinity of her capture. Green sea turtle exposure to ChePV-1 most likely occurred in the intertidal flats via contact with avian feces and feather debris. The authors consider viral penetration of keratin breaches in the green sea turtle the most likely route of transmission, similar to that seen in cutaneous poxvirus infection in farmed saltwater crocodiles [63]; other less likely routes include contact with contaminated material via ingestion, inhalation and mosquito vectors [64]. Given the unprecedent nature of avipoxvirus in a reptile, anthropogenic pressures that may have affected host immunocompetence should also be considered. In this case, the construction of three liquid natural gas (LNG) terminals on Curits Island and associated channel dredging in 2011 may have interrupted both sea turtle and migratory bird habitat use and increased the utilization of a nearby habitat, such as where this turtle was found. Additionally, increased stress in avian species may increase viral shedding [65].

The significance of ChePV-1 within the context of green sea turtle conservation is unknown. Whilst this turtle did have profound systemic pathology on necropsy and histology, this may have been caused by an unrelated virus, parasite or toxicity. Even so, immunomodulating effects of ChePV-1 predisposing this turtle to more serious disease, and vice versa, should be considered. This may be particularly relevant when considering fibropapillomatosis; as speculated previously, expression of this disease may involve complex interactions between multiple viruses, for example co-infection with chelonid herpesvirus, papillomavirus and ChePV-1. Whilst there were no cutaneous nodules associated with the ChePV-1 infection, it is unclear whether the dark purple discoloration and edema of the skin were related to the infection or were actually secondary to hypoxia and severe vasculitis. Furthermore, out of three skin samples collected from the fore flipper, type A inclusions were seen in the keratinocytes of only one 1 cm section; this may indicate that viral replication is limited in green sea turtles, not reaching levels sufficient for transmission to other individuals. In other words, they may simply act as “dead end” hosts. Alternatively, it may be that due to the lack of gross and histological changes, cheloniid poxvirus has simply gone undetected despite being endemic in green sea turtles in Gladstone Harbor, or even further afield [66].

## 5. Conclusions

This study reports evidence of a novel poxvirus, cheloniid poxvirus 1, infection in the endangered green sea turtle. The ChePV-1 genome sequence recovered was sufficiently divergent to be considered a novel avipoxvirus species and provided insight into overall genome architecture. This discovery has enhanced our understanding of the pathogen landscape relevant to green sea turtles in eastern Australia, with potential clinical and subclinical relevance to this species. However, further investigation is necessary to elucidate the host–pathogen dynamics of this disease, including the route of transmission, relevant environmental pressures driving infection, associated disease states and population prevalence.

## Figures and Tables

**Table 1:** Related poxvirus genome sequences used in further analysis for ChePV-1.

Virus	Acronym	GenBank Accession Number	Reference
canarypox virus	CNPV	AY318871	[45]
shearwaterpox virus-1	SWPV1	KX857216	[29]
shearwaterpox virus-2	SWPV2	KX857215	[29]
pigeonpox virus	FeP2	KJ801920	[46]
fowlpox virus	FWPV	AF198100 MF766430-32 MH709124-25 MH719203 MH734528 AJ581527	[47,48,49]
turkeypox virus	TKPV	NC_028238	[50]
penguinpox virus	PEPV	KJ859677	[46]
flamingopox virus	FGPV	MF678796	[51]
magpiepox virus	MPPV	MK903864	[28]
nile crocodile poxvirus	CRV	DQ356948	[19]
saltwater crocodile poxvirus-1	SwCRV1	MG450915	[20,21]
saltwater crocodile poxvirus-2	SwCRV2	MG450916	[20,21]

## Data Availability

The sequence data have been submitted to the DDBJ/EMBL/GenBank databases under accession number MT799800. Addresses are as follows: GenBank http://www.ncbi.nlm.nih.gov.

## Supplementary Materials

The following are available online at https://www.mdpi.com/1999-4915/13/2/219/s1, Table S1. Cheloniid poxvirus 1 (ChePV-1) genome annotation and comparative analysis of the predicted ORFs.
