# Supplementary material for: Emergence of a Novel Pathogenic Poxvirus Infection in the Endangered Green Sea Turtle (Chelonia mydas) Highlights a Key Threatening Process
Source: Viruses. 2021 Jan 31;13(2):219. doi: 10.3390/v13020219 (PMC7911307; doi:10.3390/v13020219)
Supplement: Supplementary file 1 [file viruses-13-00219-s001.pdf]

viruses

MDPI

Supplementary File

Emergence of a Novel Pathogenic Poxvirus Infection in the Endangered Green Sea Turtle (*Chelonia mydas*) Highlights a Key Threatening Process

Subir Sarker[1,*,†], Christabel Hannon[2,†], Ajani Athukorala[1] and Helle Bielefeldt-Ohmann[2,3]

[1] Department of Physiology, Anatomy and Microbiology, School of Life Sciences, La Trobe University, Melbourne, VIC 3086, Australia. (S.S. email: S.Sarker@latrobe.edu.au; A.A. email: a.athukorala@latrobe.edu.au).

[2] School of Veterinary Science, University of Queensland, UQ Gatton Campus, Qld 4343, Australia. (C. H. email: c.hannon1@uq.edu.au; H.B.O. email: h.bielefeldtohmann1@uq.edu.au).

[3] Australian Infectious Diseases Research Centre, The University of Queensland, St Lucia, Qld 4072, Australia

* Correspondence: S.Sarker@latrobe.edu.au; Tel.: +61 3 9479 2317; fax: (+61) 3 9479 1222

† Equal first authorship

Table S1. Cheloniid poxvirus 1 (ChePV-1) genome annotation and comparative analysis of the predicted ORFs

ChePV1 synteny	ChePV1 genome coordinates	ChePV1 AA size	SWPV2 AA size	SWPV2 synteny	SWPV2 BLAST hits	% AA identity	Notes
ChePV1-001	1481-1996	171	171	SWPV2-001	SWPV2-001 hypothetical protein	99.42	identical to ChePV1-329
ChePV1-002	2842-2216	208	208	SWPV2-002	SWPV2-002 C-type lectin-like protein	99.52	identical to ChePV1-328
ChePV1-003	3129-2923	68				93.85	CNPV004 ankyrin repeat protein, identical to ChePV1-327
ChePV1-004	3255-3923	222	222	SWPV2-003	SWPV2-003 conserved hypothetical protein	90.54	identical to ChePV1-326
ChePV1-005	***3940-4149***	***69***	***134***	***SWPV2-004***	***SWPV2-004 conserved hypothetical protein***	***93.65***	
ChePV1-006	5385-4462	307				98.37	CNPV007 ankyrin repeat protein
ChePV1-007	5970-5461	169	169	SWPV2-005	SWPV2-005 C-type lectin-like protein	100.00	
ChePV1-008	8324-6258	688	688	SWPV2-006	SWPV2-006 ankyrin repeat protein	100.00	
ChePV1-009	9324-8680	214				99.53	CNPV010 ankyrin repeat protein
ChePV1-010	11760-10000	586	586	SWPV2-007	SWPV2-007 ankyrin repeat protein	100.00	
ChePV1-011	12025-12594	189	189	SWPV2-008	SWPV2-008 conserved hypothetical protein	100.00	
ChePV1-012	13307-12801	168	168	SWPV2-009	SWPV2-009 conserved hypothetical protein	100.00	
ChePV1-013	15097-13625	490	490	SWPV2-010	SWPV2-010 Ig-like domain protein	100.00	
ChePV1-014	15261-16847	528	528	SWPV2-011	SWPV2-011 ankyrin repeat protein	100.00	
ChePV1-015	16908-17414	168	168	SWPV2-012	SWPV2-012 C-type lectin-like protein	81.55	
ChePV1-016	17518-18957	479	479	SWPV2-013	SWPV2-013 ankyrin repeat protein	75.57	
ChePV1-017	19628-19056	190	190	SWPV2-014	SWPV2-014 IL-10-like protein	100.00	
ChePV1-018	21055-19745	436	436	SWPV2-015	SWPV2-015 ankyrin repeat protein	80.50	
ChePV1-019	21235-22494	419	419	SWPV2-016	SWPV2-016 ankyrin repeat protein	99.05	
ChePV1-020	24237-22630	535	535	SWPV2-017	SWPV2-017 ankyrin repeat protein	71.78	
ChePV1-021	25354-24278	358	358	SWPV2-018	SWPV2-018 putative serpin	80.73	

ChePV1 synteny	ChePV1 genome coordinates	ChePV1 AA size	SWPV2 AA size	SWPV2 synteny	SWPV2 BLAST hits	% AA identity	Notes
ChePV1-022	26710-25454	418	424	SWPV2-019	SWPV2-019 vaccinia C4L/C10L-like protein	99.76	
ChePV1-023	26988-27524	178	178	SWPV2-020	SWPV2-020 hypothetical protein	99.44	
ChePV1-024	28640-27738	300	300	SWPV2-021	SWPV2-021 alpha-SNAP-like protein	100.00	
ChePV1-025	29880-28735	381	382	SWPV2-022	SWPV2-022 ankyrin repeat protein	80.21	
ChePV1-026	31829-29949	626	626	SWPV2-023	SWPV2-023 ankyrin repeat protein	89.94	
ChePV1-027	33045-31948	365	365	SWPV2-024	SWPV2-024 ankyrin repeat protein	97.53	
ChePV1-028	33583-33158	141	142	SWPV2-025	SWPV2-025 C-type lectin-like protein	68.09	
ChePV1-029	34659-33649	336	340	SWPV2-026	SWPV2-026 ankyrin repeat protein	94.28	
ChePV1-030	34864-35232	122	119	SWPV2-027	SWPV2-027 hypothetical protein	91.60	
ChePV1-031	36159-35428	243	242	SWPV2-028	SWPV2-028 Ig-like domain putative IFN-gamma binding protein	84.36	
ChePV1-032	36957-36223	244	246	SWPV2-029	SWPV2-029 Ig-like domain protein	82.93	
ChePV1-033	39026-37032	664	659	SWPV2-030	SWPV2-030 ankyrin repeat protein	80.73	
ChePV1-034	39647-39246	133	133	SWPV2-031	SWPV2-031 C-type lectin-like protein	78.95	
ChePV1-035	40031-39744	95	95	SWPV2-032	SWPV2-032 conserved hypothetical protein	94.74	
ChePV1-036	40095-40634	179	179	SWPV2-033	SWPV2-033 conserved hypothetical protein	61.36	
ChePV1-037	41880-40639	413	413	SWPV2-034	SWPV2-034 vaccinia C4L/C10L-like protein	94.19	
ChePV1-038	41998-42981	327	327	SWPV2-035	SWPV2-035 G protein-coupled receptor-like protein	100.00	
ChePV1-039	44775-43000	591	591	SWPV2-036	SWPV2-036 ankyrin repeat protein	83.59	
ChePV1-040	46140-44848	430	430	SWPV2-037	SWPV2-037 ankyrin repeat protein	81.82	
ChePV1-041	48009-46189	606	605	SWPV2-038	SWPV2-038 ankyrin repeat protein	75.08	
ChePV1-042	48717-48112	201	201	SWPV2-039	SWPV2-039 conserved hypothetical protein	72.64	
ChePV1-043	50201-48759	480	480	SWPV2-040	SWPV2-040 ankyrin repeat protein	72.92	
ChePV1-044	50467-51465	332	332	SWPV2-041	SWPV2-041 G protein-coupled receptor-like protein	78.61	

viruses MDPI

ChePV1 synteny	ChePV1 genome coordinates	ChePV1 AA size	SWPV2 AA size	SWPV2 synteny	SWPV2 BLAST hits	% AA identity	Notes
ChePV1-045	52844-51492	450	450	SWPV2-042	SWPV2-042 ankyrin repeat protein	75.33	
ChePV1-046	53285-52911	124	124	SWPV2-043	SWPV2-043 conserved hypothetical protein	100.00	
ChePV1-047	55858-53453	801	801	SWPV2-044	SWPV2-044 alkaline phosphodiesterase-like protein	86.14	
ChePV1-048	56398-55946	150	150	SWPV2-045	SWPV2-045 hypothetical protein	100.00	
ChePV1-049	57510-56452	352	352	SWPV2-046	SWPV2-046 ankyrin repeat protein	83.52	
ChePV1-050	58783-57557	408	408	SWPV2-047	SWPV2-047 DNase II-like protein	81.62	
ChePV1-051	59324-58809	171	171	SWPV2-048	SWPV2-048 C-type lectin-like protein	100.00	
ChePV1-052	59944-59504	146	146	SWPV2-049	SWPV2-049 conserved hypothetical protein	69.86	
ChePV1-053	60902-59937	321	140	SWPV2-050	SWPV2-050 conserved hypothetical protein	85.00	
ChePV1-054	61336-60899	145	145	SWPV2-052	SWPV2-052 dUTPase	78.62	
ChePV1-055	62283-61363	306	306	SWPV2-053	SWPV2-053 putative serpin	80.72	
ChePV1-056	62856-62314	180	180	SWPV2-054	SWPV2-054 bcl-2 like protein	76.11	
ChePV1-057	63929-62913	338	338	SWPV2-055	SWPV2-055 putative serpin	70.12	
ChePV1-058	64613-63993	206	206	SWPV2-056	SWPV2-056 conserved hypothetical protein	100.00	
ChePV1-059	66400-64697	567	565	SWPV2-057	SWPV2-057 DNA ligase	83.66	
ChePV1-060	67491-66439	350	350	SWPV2-058	SWPV2-058 putative serpin	90.00	
ChePV1-061	68638-67562	358	358	SWPV2-059	SWPV2-059 hydroxysteroid dehydrogenase-like protein	78.49	
ChePV1-062	69551-68700	283	283	SWPV2-060	SWPV2-060 TGF-beta-like protein	100.00	
ChePV1-063	71382-69631	583	583	SWPV2-061	SWPV2-061 semaphorin-like protein	92.97	
ChePV1-064	***72204-71482***	***240***	***399***	***SWPV2-062***	***SWPV2-062 hypothetical protein***	***75.35***	
ChePV1-065	72513-72340	57	57	SWPV2-063	SWPV2-063 hypothetical protein	98.25	
ChePV1-066	72673-73446	257	257	SWPV2-064	SWPV2-064 GNS1/SUR4-like protein	83.66	
ChePV1-067	73504-73971	155	155	SWPV2-065	SWPV2-065 late transcription factor VLTF-2	84.52	

**ChePV1 synteny**	**ChePV1 genome coordinates**	**ChePV1 AA size**	**SWPV2 AA size**	**SWPV2 synteny**	**SWPV2 BLAST hits**	**% AA identity**	**Notes**
ChePV1-068	73988-75643	551	551	SWPV2-066	SWPV2-066 putative rifampicin resistance protein, IMV assembly	92.01	
ChePV1-069	75675-76544	289	289	SWPV2-067	SWPV2-067 mRNA capping enzyme small subunit	90.66	
ChePV1-070	76565-76960	131	132	SWPV2-068	SWPV2-068 CC chemokine-like protein	96.97	
ChePV1-071	77902-77573	109	109	SWPV2-069	SWPV2-069 hypothetical protein	78.90	
ChePV1-072	77973-79880	635	635	SWPV2-070	SWPV2-070 NPH-I, transcription terminationfactor	92.11	
ChePV1-073	80563-79877	228	228	SWPV2-071	SWPV2-071 mutT motif putative gene expression regulator	100.00	
ChePV1-074	81245-80547	232	232	SWPV2-072	SWPV2-072 mutT motif	81.90	
ChePV1-075	83340-82858	160	160	SWPV2-073	SWPV2-073 RNA polymerase subunit RPO18	85.62	
ChePV1-076	84500-83676	274	274	SWPV2-074	SWPV2-074 Ig-like domain protein	97.08	
ChePV1-077	86524-84623	633	633	SWPV2-075	SWPV2-075 early transcription factor smallsubunit VETFS	97.31	
ChePV1-078	87710-86706	334	334	SWPV2-076	SWPV2-076 Ig-like domain protein	74.85	
ChePV1-079	90388-88004	794	794	SWPV2-077	SWPV2-077 NTPase, DNA replication	89.80	
ChePV1-080	91208-90543	221	221	SWPV2-078	SWPV2-078 CC chemokine-like protein	98.64	
ChePV1-081	91947-91291	218	218	SWPV2-079	SWPV2-079 uracil DNA glycosylase	88.99	
ChePV1-082	93198-92287	303	303	SWPV2-080	SWPV2-080 putative RNA phosphatase	83.50	
ChePV1-083	***93308-93535***	***75***	***112***	***SWPV2-081***	***SWPV2-081 TNFR-like protein***	***92.00***	
ChePV1-084	93761-94210	149	131	SWPV2-082	SWPV2-082 putative glutathione peroxidase	85.71	
ChePV1-085	94616-94068	182	159	SWPV2-084	SWPV2-084 conserved hypothetical protein	92.45	
ChePV1-086	94986-94603	127	127	SWPV2-085	SWPV2-085 conserved hypothetical protein	73.23	
ChePV1-087	***95308-95072***	***78***	***83***	***SWPV2-086***	***SWPV2-086 HT motif protein***	***85.90***	
ChePV1-088	***96089-95805***	***94***	***146***	***SWPV2-087***	***SWPV2-087 conserved hypothetical protein***	***88.04***	
ChePV1-089	96994-96191	267	267	SWPV2-088	SWPV2-088 virion protein	90.87	

ChePV1 synteny	ChePV1 genome coordinates	ChePV1 AA size	SWPV2 AA size	SWPV2 synteny	SWPV2 BLAST hits	% AA identity	Notes
ChePV1-090	97069-97893	274	275	SWPV2-089	SWPV2-089 T10-like protein	85.40	
ChePV1-091	98041-97904	45	45	SWPV2-090	SWPV2-090 conserved hypothetical protein	100.00	
ChePV1-092	98280-98023	85	85	SWPV2-091	SWPV2-091 ubiquitin	100.00	
ChePV1-093	99407-98394	337	339	SWPV2-092	SWPV2-092 conserved hypothetical protein	83.78	
ChePV1-094	99670-99428	80	80	SWPV2-093	SWPV2-093 hypothetical protein	97.50	
ChePV1-095	100263-99676	195	195	SWPV2-094	SWPV2-094 beta-NGF-like protein	97.44	
ChePV1-096	100793-100287	168	168	SWPV2-095	SWPV2-095 putative interleukin binding protein	100.00	
ChePV1-097	101105-100848	85	85	SWPV2-096	SWPV2-096 hypothetical protein	61.18	
ChePV1-098	101433-101116	105	105	SWPV2-097	SWPV2-097 conserved hypothetical protein	55.24	
ChePV1-099	102022-101450	190	190	SWPV2-098	SWPV2-098 N1R/p28-like protein	82.11	
ChePV1-100	102224-102601	125	125	SWPV2-099	SWPV2-099 putative glutaredoxin 2, virion morphogenesis	83.20	
ChePV1-101	103248-102544	234	234	SWPV2-100	SWPV2-100 putative elongation factor	76.07	
ChePV1-102	103242-103550	102	102	SWPV2-101	SWPV2-101 conserved hypothetical protein	78.05	
ChePV1-103	103686-103919	77	77	SWPV2-102	SWPV2-102 hypothetical protein	100.00	
ChePV1-104	104164-106062	632	632	SWPV2-103	SWPV2-103 putative metalloprotease, virionmorphogenesis	88.92	
ChePV1-105	108091-106046	681	681	SWPV2-104	SWPV2-104 NPH-II, RNA helicase	87.22	
ChePV1-106	108126-109394	422	422	SWPV2-105	SWPV2-105 virion core proteinase	87.68	
ChePV1-107	109399-110574	391	391	SWPV2-106	SWPV2-106 DNA-binding protein	84.91	
ChePV1-108	110575-110820	81	81	SWPV2-107	SWPV2-107 putative IMV membrane protein	71.60	
ChePV1-109	110842-111381	179	179	SWPV2-108	SWPV2-108 thymidine kinase	75.98	
ChePV1-110	111502-111750	82	82	SWPV2-109	SWPV2-109 HT motif protein	95.12	
ChePV1-111	111820-112689	289	289	SWPV2-110	SWPV2-110 DNA-binding phosphoprotein	71.97	
ChePV1-112	112690-112899	69	69	SWPV2-111	SWPV2-111 conserved hypothetical protein	79.71	

ChePV1 synteny	ChePV1 genome coordinates	ChePV1 AA size	SWPV2 AA size	SWPV2 synteny	SWPV2 BLAST hits	% AA identity	Notes
ChePV1-113	112906-113838	310	310	SWPV2-112	SWPV2-112 DNA-binding virion protein	92.26	
ChePV1-114	114018-115964	648	652	SWPV2-113	SWPV2-113 conserved hypothetical protein	77.16	
ChePV1-115	115906-116301	131	131	SWPV2-114	SWPV2-114 virion core protein	77.86	
ChePV1-116	116579-116298	93	93	SWPV2-115	SWPV2-115 putative IMV redox protein, virus assembly	86.96	
ChePV1-117	116606-119572	988	988	SWPV2-116	SWPV2-116 DNA polymerase	88.56	
ChePV1-118	119911-119564	115				78.15	SWPV1-111 putative membrane protein
ChePV1-119	120252-119989	87				88.24	SWPV1-111 putative membrane protein
ChePV1-120	121935-120427	502	502	SWPV2-117	SWPV2-117 conserved hypothetical protein	82.27	
ChePV1-121	***124978-121997***	***993***	***1916***	***SWPV2-118***	***SWPV2-118 variola B22R-like protein***	***93.96***	
ChePV1-122	***127709-125349***	***786***	***1916***	***SWPV2-118***	***SWPV2-118 variola B22R-like protein***	***79.87***	
ChePV1-123	133079-127776	1767	1767	SWPV2-119	SWPV2-119 variola B22R-like protein	87.44	
ChePV1-124	138878-133359	1839	1839	SWPV2-120	SWPV2-120 variola B22R-like protein	85.10	
ChePV1-125	139200-138739	153	153	SWPV2-121	SWPV2-121 variola B22R-like protein	98.69	
ChePV1-126	139290-139838	182	182	SWPV2-122	SWPV2-122 RNA polymerase subunit RPO30	98.90	
ChePV1-127	139870-142044	724	721	SWPV2-123	SWPV2-123 conserved hypothetical protein	82.25	
ChePV1-128	142028-143446	472	472	SWPV2-124	SWPV2-124 poly(A) polymerase large subunit PAPL	87.92	
ChePV1-129	143799-143440	119	119	SWPV2-125	SWPV2-125 DNA-binding virion core protein	84.03	
ChePV1-130	143875-144498	207	207	SWPV2-126	SWPV2-126 conserved hypothetical protein	98.55	
ChePV1-131	144592-145038	148	148	SWPV2-127	SWPV2-127 conserved hypothetical protein	97.30	
ChePV1-132	145272-145571	99	99	SWPV2-128	SWPV2-128 conserved hypothetical protein	84.85	
ChePV1-133	151047-145642	1801	1801	SWPV2-129	SWPV2-129 variola B22R-like protein	84.18	
ChePV1-134	151198-151037	53					hypothetical protein, unique to ChePV1,

ChePV1 synteny	ChePV1 genome coordinates	ChePV1 AA size	SWPV2 AA size	SWPV2 synteny	SWPV2 BLAST hits	% AA identity	Notes
							detected one transmembrane helix
ChePV1-135	151207-152343	378	378	SWPV2-130	SWPV2-130 putative palmitylated EEV envelope lipase	89.68	
ChePV1-136	152421-154298	625	625	SWPV2-131	SWPV2-131 putative EEV maturation protein	88.96	
ChePV1-137	154341-155729	462	462	SWPV2-132	SWPV2-132 conserved hypothetical protein	77.27	
ChePV1-138	155820-157154	444	444	SWPV2-133	SWPV2-133 putative serine/threonine protein kinase, virus assembly	86.94	
ChePV1-139	157129-157770	213	213	SWPV2-134	SWPV2-134 conserved hypothetical protein	85.92	
ChePV1-140	157853-158053	66	66	SWPV2-135	SWPV2-135 conserved hypothetical protein	80.30	
ChePV1-141	158379-158933	184	184	SWPV2-136	SWPV2-136 HAL3-like domain protein	88.59	
ChePV1-142	159194-160159	321	321	SWPV2-137	SWPV2-137 N1R/p28-like protein	88.16	
ChePV1-143	160271-162310	679	671	SWPV2-138	SWPV2-138 ankyrin repeat protein	95.22	
ChePV1-144	162312-163982	556	556	SWPV2-139	SWPV2-139 ankyrin repeat protein	98.02	
ChePV1-145	164203-165525	440	440	SWPV2-140	SWPV2-140 conserved hypothetical protein	90.91	
ChePV1-146	165533-165721	62	62	SWPV2-141	SWPV2-141 RNA polymerase subunit RPO7	100.00	
ChePV1-147	165714-166280	188	188	SWPV2-142	SWPV2-142 conserved hypothetical protein	85.11	
ChePV1-148	167291-166245	348	348	SWPV2-143	SWPV2-143 virion core protein	85.06	
ChePV1-149	168377-167457	306	306	SWPV2-144	SWPV2-144 putative thioredoxin binding protein	100.00	
ChePV1-150	***168505-168750***	***81***	***412***	***SWPV2-145***	***SWPV2-145 ankyrin repeat protein***	***40.82***	
ChePV1-151	170104-168866	412	412	SWPV2-145	SWPV2-145 ankyrin repeat protein	100.00	
ChePV1-152	170780-170331	149	149	SWPV2-146	SWPV2-146 hypothetical protein	100.00	
ChePV1-153	171946-171008	312	312	SWPV2-147	SWPV2-147 Rep-like protein	100.00	
ChePV1-154	***175364-172380***	***994***	***1831***	***SWPV2-149***	***SWPV2-149 variola B22R-like protein***	***50.35***	
ChePV1-155	178158-175531	875	875	SWPV2-148	SWPV2-148 variola B22R-like protein	88.15	
ChePV1-156	183706-178211	1831	1831	SWPV2-149	SWPV2-149 variola B22R-like protein	84.34	

ChePV1 synteny	ChePV1 genome coordinates	ChePV1 AA size	SWPV2 AA size	SWPV2 synteny	SWPV2 BLAST hits	% AA identity	Notes
ChePV1-157	***184023-184490***	***155***	***834***	***SWPV2-150***	***SWPV2-150 hypothetical protein***	***85.94***	
ChePV1-158	***184499-185989***	***496***	***834***	***SWPV2-150***	***SWPV2-150 hypothetical protein***	***94.86***	
ChePV1-159	***185916-186455***	***179***	***834***	***SWPV2-150***	***SWPV2-150 hypothetical protein***	***100.00***	
ChePV1-160	187583-186552	343	343	SWPV2-151	SWPV2-151 TGF-beta-like protein	100.00	
ChePV1-161	187807-188055	82	149	SWPV2-153	SWPV2-153 TGF-beta-like protein	49.38	
ChePV1-162	188040-188315	91	85	SWPV2-208	SWPV2-208 N1R/p28-like protein	68.60	
ChePV1-163	189167-189451	94	94	SWPV2-213	SWPV2-213 N1R/p28-like protein	61.11	
ChePV1-164	***189426-190262***	***278***	***350***	***SWPV2-157***	***SWPV2-157 N1R/p28-like protein***	***80.00***	
ChePV1-165	191437-190358	359	358	SWPV2-152	SWPV2-152 TGF-beta-like protein	75.14	
ChePV1-166	191487-191936	149	149	SWPV2-153	SWPV2-153 TGF-beta-like protein	83.89	
ChePV1-167	192354-193316	320	320	SWPV2-154	SWPV2-154 N1R/p28-like protein	92.19	
ChePV1-168	193551-194585	344	345	SWPV2-155	SWPV2-155 Ig-like domain protein	77.91	
ChePV1-169	***195275-195943***	***222***	***350***	***SWPV2-157***	***SWPV2-157 N1R/p28-like protein***	***67.30***	
ChePV1-170	***196492-197160***	***222***	***320***	***SWPV2-154***	***SWPV2-154 N1R/p28-like protein***	***72.45***	
ChePV1-171	197233-197871	212	212	SWPV2-158	SWPV2-158 thymidylate kinase	99.53	
ChePV1-172	197924-198706	260	260	SWPV2-159	SWPV2-159 late transcription factor VLTF-1	98.85	
ChePV1-173	198720-199727	335	335	SWPV2-160	SWPV2-160 putative myristylated protein	91.22	
ChePV1-174	199728-200459	243	243	SWPV2-161	SWPV2-161 putative myristylated IMV envelope protein	93.00	
ChePV1-175	200519-200809	96	96	SWPV2-162	SWPV2-162 conserved hypothetical protein	98.96	
ChePV1-176	201710-200799	303	303	SWPV2-163	SWPV2-163 conserved hypothetical protein	85.48	
ChePV1-177	201736-202494	252	252	SWPV2-164	SWPV2-164 DNA-binding virion core protein	88.49	
ChePV1-178	202495-202887	130	130	SWPV2-165	SWPV2-165 conserved hypothetical protein	82.31	
ChePV1-179	202841-203287	148	148	SWPV2-166	SWPV2-166 putative IMV membrane protein	89.86	

ChePV1 synteny	ChePV1 genome coordinates	ChePV1 AA size	SWPV2 AA size	SWPV2 synteny	SWPV2 BLAST hits	% AA identity	Notes
ChePV1-180	203321-204229	302	302	SWPV2-167	SWPV2-167 poly(A) polymerase small subunit PAPS	89.63	
ChePV1-181	204226-204786	186	186	SWPV2-168	SWPV2-168 RNA polymerase subunit RPO22	88.17	
ChePV1-182	205189-204779	136	136	SWPV2-169	SWPV2-169 conserved hypothetical protein	87.50	
ChePV1-183	205232-209098	1288	1288	SWPV2-170	SWPV2-170 RNA polymerase subunit RPO147	94.88	
ChePV1-184	209601-209101	166	166	SWPV2-171	SWPV2-171 putative protein-tyrosine phosphatase, virus assembly	88.55	
ChePV1-185	209617-210186	189	189	SWPV2-172	SWPV2-172 conserved hypothetical protein	88.89	
ChePV1-186	211248-210262	328	328	SWPV2-173	SWPV2-173 ankyrin repeat protein	99.70	
ChePV1-187	212283-211291	330	330	SWPV2-174	SWPV2-174 putative IMV envelope protein	80.91	
ChePV1-188	214774-212375	799	799	SWPV2-175	SWPV2-175 RNA polymerase associated protein RAP94	92.12	
ChePV1-189	214943-215455	170	170	SWPV2-176	SWPV2-176 late transcription factor VLTF-4	89.41	
ChePV1-190	215456-216406	316	316	SWPV2-177	SWPV2-177 DNA topoisomerase	89.87	
ChePV1-191	216411-216872	153	153	SWPV2-178	SWPV2-178 conserved hypothetical protein	82.35	
ChePV1-192	217146-216835	103	103	SWPV2-179	SWPV2-179 conserved hypothetical protein	68.93	
ChePV1-193	217154-219694	846	846	SWPV2-180	SWPV2-180 mRNA capping enzyme large subunit	89.95	
ChePV1-194	219771-220085	104	106	SWPV2-181	SWPV2-181 HT motif protein	80.00	
ChePV1-195	220504-220082	140	140	SWPV2-182	SWPV2-182 virion protein	80.00	
ChePV1-196	220558-220992	144	144	SWPV2-183	SWPV2-183 hypothetical protein	100.00	
ChePV1-197	221057-221626	189	190	SWPV2-184	SWPV2-184 conserved hypothetical protein	73.02	
ChePV1-198	221695-222522	275	275	SWPV2-185	SWPV2-185 N1R/p28-like protein	100.00	
ChePV1-199	223059-222589	156	156	SWPV2-186	SWPV2-186 C-type lectin-like protein	100.00	
ChePV1-200	223367-224044	225	225	SWPV2-187	SWPV2-187 deoxycytidine kinase-like protein	89.33	
ChePV1-201	224050-224550	166	166	SWPV2-188	SWPV2-188 Rep-like protein	83.73	
ChePV1-202	224609-225112	167	167	SWPV2-189	SWPV2-189 conserved hypothetical protein	100.00	

ChePV1 synteny	ChePV1 genome coordinates	ChePV1 AA size	SWPV2 AA size	SWPV2 synteny	SWPV2 BLAST hits	% AA identity	Notes
ChePV1-203	225166-225996	276	276	SWPV2-190	SWPV2-190 N1R/p28-like protein	69.93	
ChePV1-204	226069-227217	382	382	SWPV2-191	SWPV2-191 N1R/p28-like protein	86.39	
ChePV1-205	227273-227458	61	61	SWPV2-192	SWPV2-192 conserved hypothetical protein	100.00	
ChePV1-206	227677-228633	318	318	SWPV2-193	SWPV2-193 N1R/p28-like protein	81.76	
ChePV1-207	228793-230112	439	472	SWPV2-194	SWPV2-194 putative photolyase	84.28	
ChePV1-208	230241-230762	173	173	SWPV2-195	SWPV2-195 N1R/p28-like protein	84.97	
ChePV1-209	230891-231493	200	200	SWPV2-196	SWPV2-196 conserved hypothetical protein	86.00	
ChePV1-210	231537-232469	310	310	SWPV2-197	SWPV2-197 N1R/p28-like protein	76.77	
ChePV1-211	232517-232912	131	131	SWPV2-198	SWPV2-198 N1R/p28-like protein	100.00	
ChePV1-212	232967-233131	54	54	SWPV2-199	SWPV2-199 conserved hypothetical protein	100.00	
ChePV1-213	233191-233721	176	176	SWPV2-200	SWPV2-200 N1R/p28-like protein	77.59	
ChePV1-214	234415-233765	216	216	SWPV2-201	SWPV2-201 deoxycytidine kinase-like protein	90.74	
ChePV1-215	234589-235659	356	356	SWPV2-202	SWPV2-202 vaccinia C4L/C10L-like protein	100.00	
ChePV1-216	235934-236548	204	204	SWPV2-203	SWPV2-203 CC chemokine-like protein	100.00	
ChePV1-217	236638-237843	401	401	SWPV2-204	SWPV2-204 conserved hypothetical protein	100.00	
ChePV1-218	237938-238930	330	330	SWPV2-205	SWPV2-205 N1R/p28-like protein	100.00	
ChePV1-219	239018-239689	223	223	SWPV2-206	SWPV2-206 N1R/p28-like protein	100.00	
ChePV1-220	240590-240393	65					hypothetical protein, unique to ChePV1, detected one transmembrane helix
ChePV1-221	241141-242190	349	349	SWPV2-207	SWPV2-207 N1R/p28-like protein	98.85	
ChePV1-222	242216-242476	86	349	SWPV2-207	SWPV2-207 N1R/p28-like protein	66.27	
ChePV1-223	242547-243188	213	213	SWPV2-209	SWPV2-209 N1R/p28-like protein	89.20	
ChePV1-224	243656-244513	285	285	SWPV2-210	SWPV2-210 N1R/p28-like protein	99.65	

ChePV1 synteny	ChePV1 genome coordinates	ChePV1 AA size	SWPV2 AA size	SWPV2 synteny	SWPV2 BLAST hits	% AA identity	Notes
ChePV1-225	247575-245032	847	847	SWPV2-211	SWPV2-211 ankyrin repeat protein	99.76	
ChePV1-226	247829-248548	239	239	SWPV2-212	SWPV2-212 hypothetical protein	100.00	
ChePV1-227	248876-248712	54					hypothetical protein, unique to ChePV1, detected two transmembrane helices
ChePV1-228	249159-249704	181	126	SWPV2-214	SWPV2-214 N1R/p28-like protein	82.26	
ChePV1-229	251630-250326	434	434	SWPV2-215	SWPV2-215 ankyrin repeat protein	100.00	
ChePV1-230	251828-252025	65	65	SWPV2-216	SWPV2-216 hypothetical protein	98.46	
ChePV1-231	251973-252449	158	158	SWPV2-217	SWPV2-217 MyD116-like domain protein	100.00	
ChePV1-232	252479-253093	204	204	SWPV2-218	SWPV2-218 CC chemokine-like protein	99.02	
ChePV1-233	253233-254648	471	471	SWPV2-219	SWPV2-219 ankyrin repeat protein	100.00	
ChePV1-234	254668-256032	454	508	SWPV2-220	SWPV2-220 ankyrin repeat protein	66.08	
ChePV1-235	256265-257563	432	432	SWPV2-221	SWPV2-221 conserved hypothetical protein	99.08	
ChePV1-236	257608-258579	323	323	SWPV2-222	SWPV2-222 ribonucleotide reductase small subunit	87.62	
ChePV1-237	258760-260085	441	441	SWPV2-223	SWPV2-223 ankyrin repeat protein	99.32	
ChePV1-238	260801-260124	225	225	SWPV2-224	SWPV2-224 late transcription factor VLTF-3	95.56	
ChePV1-239	261016-260798	72	75	SWPV2-225	SWPV2-225 virion redox protein	94.44	
ChePV1-240	263009-261030	659	659	SWPV2-226	SWPV2-226 virion core protein P4b	89.53	
ChePV1-241	263743-263096	215	215	SWPV2-227	SWPV2-227 immunodominant virion protein	81.86	
ChePV1-242	263782-264291	169	169	SWPV2-228	SWPV2-228 RNA polymerase subunit RPO19	91.72	
ChePV1-243	265407-264286	373	373	SWPV2-229	SWPV2-229 conserved hypothetical protein	85.25	
ChePV1-244	267543-265414	709	709	SWPV2-230	SWPV2-230 early transcription factor large subunit VETFL	94.78	
ChePV1-245	267607-268509	300	300	SWPV2-231	SWPV2-231 intermediate transcription factor VITF-3	91.67	

ChePV1 synteny	ChePV1 genome coordinates	ChePV1 AA size	SWPV2 AA size	SWPV2 synteny	SWPV2 BLAST hits	% AA identity	Notes
ChePV1-246	268701-268471	76	75	SWPV2-232	SWPV2-232 putative IMV membrane protein	81.33	
ChePV1-247	271383-268702	893	893	SWPV2-233	SWPV2-233 virion core protein P4a	84.10	
ChePV1-248	271401-272240	279	279	SWPV2-234	SWPV2-234 conserved hypothetical protein	88.53	
ChePV1-249	272743-272237	168	168	SWPV2-235	SWPV2-235 virion protein	86.23	
ChePV1-250	272758-272952	64	56	SWPV2-236	SWPV2-236 conserved hypothetical protein	73.21	
ChePV1-251	273251-273042	69	69	SWPV2-237	SWPV2-237 putative IMV membrane protein	71.01	
ChePV1-252	273577-273299	92	92	SWPV2-238	SWPV2-238 putative IMV membrane protein	67.39	
ChePV1-253	273755-273594	53	53	SWPV2-239	SWPV2-239 putative IMV membrane virulence factor	75.47	
ChePV1-254	274061-273771	96	96	SWPV2-240	SWPV2-240 conserved hypothetical protein	75.00	
ChePV1-255	275151-274045	368	368	SWPV2-241	SWPV2-241 predicted myristylated protein	87.23	
ChePV1-256	275745-275167	192	192	SWPV2-242	SWPV2-242 putative phosphorylated IMV membrane protein	94.59	
ChePV1-257	275763-277151	462	462	SWPV2-243	SWPV2-243 DNA helicase, transcriptional elongation	88.74	
ChePV1-258	277388-277119	89	89	SWPV2-244	SWPV2-244 conserved hypothetical protein	80.49	
ChePV1-259	277734-277396	112	112	SWPV2-245	SWPV2-245 conserved hypothetical protein	91.96	
ChePV1-260	277733-279028	431	434	SWPV2-246	SWPV2-246 DNA polymerase processivity factor	82.79	
ChePV1-261	279034-279492	152	152	SWPV2-247	SWPV2-247 Holliday junction resolvase protein	82.89	
ChePV1-262	279509-280660	383	383	SWPV2-248	SWPV2-248 intermediate transcription factor VITF-3	85.90	
ChePV1-263	280686-284159	1157	1157	SWPV2-249	SWPV2-249 RNA polymerase subunit RPO132	94.90	
ChePV1-264	285953-284148	601	601	SWPV2-250	SWPV2-250 A type inclusion-like protein	84.86	
ChePV1-265	287415-285988	475	475	SWPV2-251	SWPV2-251 A type inclusion-like fusion protein	96.63	
ChePV1-266	287838-287416	140	140	SWPV2-252	SWPV2-252 conserved hypothetical protein	87.86	
ChePV1-267	288760-287843	305	305	SWPV2-253	SWPV2-253 RNA polymerase subunit RPO35	79.02	
ChePV1-268	288962-288735	75	75	SWPV2-254	SWPV2-254 conserved hypothetical protein	74.67	

ChePV1 synteny	ChePV1 genome coordinates	ChePV1 AA size	SWPV2 AA size	SWPV2 synteny	SWPV2 BLAST hits	% AA identity	Notes
ChePV1-269	289087-289428	113	113	SWPV2-255	SWPV2-255 conserved hypothetical protein	76.11	
ChePV1-270	289437-289796	119	120	SWPV2-256	SWPV2-256 conserved hypothetical protein	69.75	
ChePV1-271	290642-289788	284	284	SWPV2-257	SWPV2-257 DNA packaging protein	95.07	
ChePV1-272	290757-291302	181	181	SWPV2-258	SWPV2-258 C-type lectin-like EEV protein	77.90	
ChePV1-273	291527-292351	274	274	SWPV2-259	SWPV2-259 conserved hypothetical protein	92.70	
ChePV1-274	292411-293220	269	269	SWPV2-260	SWPV2-260 putative tyrosine protein kinase	100.00	
ChePV1-275	293263-294279	338	338	SWPV2-261	SWPV2-261 putative serpin	100.00	
ChePV1-276	295059-294301	252	252	SWPV2-262	SWPV2-262 conserved hypothetical protein	96.03	
ChePV1-277	295169-296101	310	310	SWPV2-263	SWPV2-263 G protein-coupled receptor-like protein	87.10	
ChePV1-278	296112-296402	96	96	SWPV2-264	SWPV2-264 conserved hypothetical protein	87.50	
ChePV1-279	296468-296977	169	169	SWPV2-265	SWPV2-265 beta-NGF-like protein	100.00	
ChePV1-280	297387-296983	134	130	SWPV2-266	SWPV2-266 HT motif protein	73.08	
ChePV1-281	297491-298135	214	214	SWPV2-267	SWPV2-267 conserved hypothetical protein	82.71	
ChePV1-282	298508-298146	120	120	SWPV2-268	SWPV2-268 HT motif protein	72.50	
ChePV1-283	298674-299009	111	111	SWPV2-269	SWPV2-269 CC chemokine-like protein	100.00	
ChePV1-284	299081-299662	193	193	SWPV2-270	SWPV2-270 putative interleukin binding protein	97.93	
ChePV1-285	299772-300170	132	126	SWPV2-271	SWPV2-271 EGF-like protein	99.21	
ChePV1-286	300154-301071	305	305	SWPV2-272	SWPV2-272 putative serine/threonine protein kinase	81.31	
ChePV1-287	301114-301596	160	160	SWPV2-273	SWPV2-273 conserved hypothetical protein	100.00	
ChePV1-288	301632-302075	147	147	SWPV2-274	SWPV2-274 C-type lectin-like protein	76.19	
ChePV1-289	302118-302537	139	139	SWPV2-275	SWPV2-275 putative interleukin binding protein	100.00	
ChePV1-290	302606-302833	75	75	SWPV2-276	SWPV2-276 conserved hypothetical protein	89.33	
ChePV1-291	303035-304819	594	594	SWPV2-277	SWPV2-277 ankyrin repeat protein	97.81	
ChePV1-292	304843-305067	74	74	SWPV2-278	SWPV2-278 hypothetical protein	100.00	

ChePV1 synteny	ChePV1 genome coordinates	ChePV1 AA size	SWPV2 AA size	SWPV2 synteny	SWPV2 BLAST hits	% AA identity	Notes
ChePV1-293	305110-305964	284	284	SWPV2-279	SWPV2-279 ankyrin repeat protein	99.65	
ChePV1-294	306019-307311	430	430	SWPV2-280	SWPV2-280 ankyrin repeat protein	99.77	
ChePV1-295	307504-308694	396	396	SWPV2-281	SWPV2-281 ankyrin repeat protein	74.30	
ChePV1-296	***308745-310073***	***442***	***458***	***SWPV2-282***	***SWPV2-282 ankyrin repeat protein***	***69.46***	
ChePV1-297	310170-312383	737	737	SWPV2-283	SWPV2-283 ankyrin repeat protein	90.91	
ChePV1-298	312439-315060	873	571	SWPV2-284	SWPV2-284 ankyrin repeat protein	71.75	
ChePV1-299	315133-315867	244	244	SWPV2-286	SWPV2-286 ankyrin repeat protein	79.92	
ChePV1-300	316468-318051	527	527	SWPV2-287	SWPV2-287 ankyrin repeat protein	82.35	
ChePV1-301	318647-318066	193	193	SWPV2-288	SWPV2-288 conserved hypothetical protein	76.68	
ChePV1-302	318715-320217	500	500	SWPV2-289	SWPV2-289 ankyrin repeat protein	75.20	
ChePV1-303	320433-321833	466	466	SWPV2-290	SWPV2-290 ankyrin repeat protein	79.83	
ChePV1-304	321904-322692	262	262	SWPV2-291	SWPV2-291 N1R/p28-like protein	82.06	
ChePV1-305	322754-322972	72	72	SWPV2-292	SWPV2-292 hypothetical protein	97.22	
ChePV1-306	323440-322976	154	154	SWPV2-293	SWPV2-293 C-type lectin-like protein	100.00	
ChePV1-307	323623-324699	358	357	SWPV2-294	SWPV2-294 ankyrin repeat protein	63.56	
ChePV1-308	324838-325428	196	196	SWPV2-295	SWPV2-295 ankyrin repeat protein	100.00	
ChePV1-309	325533-327146	537	537	SWPV2-296	SWPV2-296 ankyrin repeat protein	88.83	
ChePV1-310	327180-327554	124	124	SWPV2-297	SWPV2-297 EFc-like protein	100.00	
ChePV1-311	327564-328064	166	166	SWPV2-298	SWPV2-298 conserved hypothetical protein	100.00	
ChePV1-312	328136-328792	218	218	SWPV2-299	SWPV2-299 Ig-like domain protein	91.74	
ChePV1-313	328819-330702	627	629	SWPV2-300	SWPV2-300 ankyrin repeat protein	75.24	
ChePV1-314	330807-331754	315	315	SWPV2-301	SWPV2-301 G protein-coupled receptor-like protein	100.00	
ChePV1-315	331821-333455	544	544	SWPV2-302	SWPV2-302 ankyrin repeat protein	100.00	
ChePV1-316	333636-333803	55	55	SWPV2-303	SWPV2-303 hypothetical protein	100.00	

ChePV1 synteny	ChePV1 genome coordinates	ChePV1 AA size	SWPV2 AA size	SWPV2 synteny	SWPV2 BLAST hits	% AA identity	Notes
ChePV1-317	333978-334742	254	514	SWPV2-304	SWPV2-304 ankyrin repeat protein	99.21	
ChePV1-318	***335071-335241***	***56***	***514***	***SWPV2-304***	***SWPV2-304 ankyrin repeat protein***	***91.07***	
ChePV1-319	335338-335907	189	124	SWPV2-307	SWPV2-307 EFc-like protein	95.48	
ChePV1-320	***335943-336176***	***77***	***637***	***SWPV2-305***	***SWPV2-305 ankyrin repeat protein***	***83.02***	
ChePV1-321	336301-337758	485	469	SWPV2-306	SWPV2-306 Ig-like domain protein	82.52	
ChePV1-322	337788-338162	124	124	SWPV2-307	SWPV2-307 EFc-like protein	79.03	
ChePV1-323	***338170-338346***	***58***	***689***	***SWPV2-308***	***SWPV2-308 ankyrin repeat protein***	***56.00***	
ChePV1-324	***338462-338677***	***71***	***689***	***SWPV2-308***	***SWPV2-308 ankyrin repeat protein***	***75.00***	
ChePV1-325	***339193-338990***	***67***	***186***	***SWPV2-309***	***SWPV2-309 conserved hypothetical protein***	***95.00***	
ChePV1-326	339878-339210	222	222	SWPV2-310	SWPV2-310 conserved hypothetical protein	90.54	identical to ChePV1-004
ChePV1-327	340004-340210	68				93.85	CNPV004 ankyrin repeat protein, identical to ChePV1-003
ChePV1-328	340291-340917	208	208	SWPV2-311	SWPV2-311 C-type lectin-like protein	99.52	identical to ChePV1-002
ChePV1-329	341652-341137	171	171	SWPV2-312	SWPV2-312 hypothetical protein	99.42	identical to ChePV1-001

The abbreviations for poxviruses were used: ChePV1, cheloniid poxvirus; SWPV1, shearwaterpox virus 1; SWPV2, shearwaterpox virus 2; CNPV, canarypox virus. Truncated or fragmented ORFs of ChePV1 were highlighted with bold and italicised font.
